# Delayed diagnosis of multiple systemic disseminated tuberculosis: A case report

**DOI:** 10.1097/MD.0000000000028656

**Published:** 2022-02-18

**Authors:** Huan Li, Fang He, Cejun Zhong, Junyan Qu

**Affiliations:** Center of Infectious Disease, West China Hospital, Sichuan University, 37 Guoxue Lane, Chengdu, China.

**Keywords:** diagnosis, disseminated tuberculosis, imaging examination

## Abstract

**Rationale:**

The clinical manifestations in patients with disseminated tuberculosis (TB) are nonspecific and may present challenges for early diagnosis.

**Patient concerns and diagnoses:**

We describe the case of a 44-year-old man who presented with abdominal pain and discomfort for more than a month. He had undergone surgery for a chest wall abscess 9 months ago. Computed tomography scans showed a miliary pattern in the lung and multiple abscesses in the liver, spleen, left psoas major muscle, skin, and soft tissue, with rim enhancement. *Mycobacterium tuberculosis* was detected in the drainage fluid of the abscesses and surgical slices, and disseminated TB was diagnosed.

**Interventions and outcomes:**

With anti-tuberculosis therapy, the abscesses were gradually absorbed and all cultures were negative.

**Lessons:**

The patient exemplifies the difficulty of the early diagnosis of disseminated TB. Disseminated TB should be considered first in patients with multisystem illness, and then evidence should be pursued relentlessly to establish a diagnosis.

## 1 Introduction

Despite significant progress in recent years, tuberculosis (TB) remains a formidable global health threat with about 10.4 million new cases and 1.8 million deaths each year.^[1]^ Disseminated TB can mimic many diseases and presents with variable and nonspecific clinical features, which may easily lead to misdiagnosis or delayed diagnosis. Late diagnosis of a TB infection and delayed anti-TB treatment are risk factors for poor prognosis of disseminated TB.^[^2^,^^3^^]^ Therefore, early diagnosis and treatment are critical.

Here, we report a case of multiple systemic disseminated TB in a patient who had been misdiagnosed with multiple abscesses for nearly 10 months. After aggressive anti-TB treatment, the foci were absorbed, and the symptoms improved. We highlight the challenges in the early diagnosis of disseminated TB and review the relevant literature.

## 2 Case presentation

A 44-year-old man was admitted to our hospital on April 9, 2019, complaining of recurrent abdominal pain for more than a month. In February 2019, the patient experienced left lower quadrant abdominal pain without any obvious incentives. An intra-abdominal abscess was diagnosed using computed tomography at a local hospital. The patient underwent mesenteric abscess drainage and enterolysis on February 18, 2019. Three drains were left in place at that time. *Streptococcus viridans* was cultured from pus and cephalosporin was administered. The patient's condition improved and he was discharged. Approximately half a month ago, he complained of recurrent abdominal pain and a subcutaneous mass under the left jaw without redness, but had no respiratory distress, fever with chills, night sweets, or diarrhea. The patient was referred to our hospital for further treatment. The patient had a history of surgery for left subclavicular and supraclavicular abscesses more than 9 months ago and perianal abscess with anal fistula 5 months ago.

**Figure 1:**
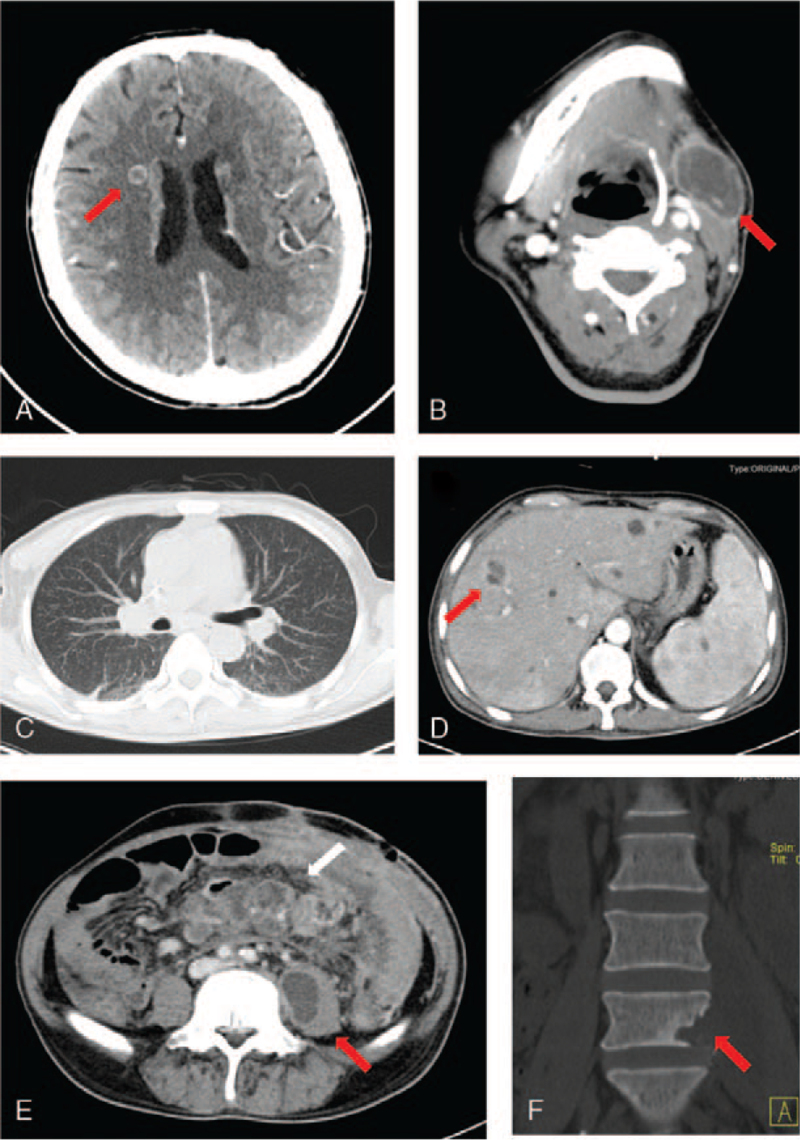
Computer tomography findings of multiple organs in this patient. Head and neck CT showing a strengthening nodule near the right lateral ventricle (A, arrow) and a mass shadow with annular enhancement under the left jaw (B, arrow). Chest computed tomography (CT) showing diffuse miliary nodules in both lungs (C). Abdominal CT reveals multiple ring-enhancing low-density nodules in the liver (D, arrow). Multiple abdominal lymph nodes, including mesenteric lymph nodes, are enlarged with an enhancing rim (E, white arrow). The left psoas major muscle is swollen with a low-density shadow and annular enhancement (E, red arrow). The left side of the L5 vertebra shows bone destruction (F).

**Figure 2:**
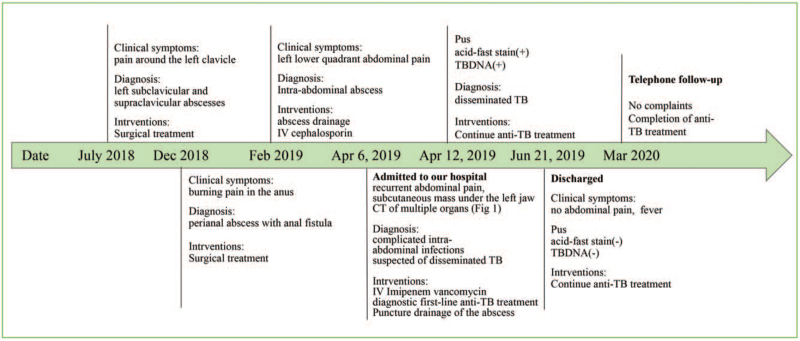
Timeline of relevant data of the diagnosis and interventions of the patient. DNA = deoxyribonucleic acid, TB = tuberculosis.

On admission, the patient was alert and his vital signs were normal. Physical examination revealed 2 unevenly old surgical scars in the supraclavicular fossa (1 × 1 cm) and subclavian fossa (5 × 4 cm). There was an increasing mass in his neck and ulceration in his left lower abdominal wall with purulent secretion. Another surgical scar (15 × 1 cm) in the middle abdomen presented with decadent purulent mucus. Two sinus tracts were formed in the lower left and right abdomen, with purulent secretions and no stench. A routine blood test revealed the following: white blood cell count, 12.05 × 10^9^/L; neutrophils, 90.1%; lymphocytes, 6.1%; hemoglobin, 96 g/L; platelets, 301 × 10^9^/L; erythrocyte sedimentation rate, 69 mm/h; C-reactive protein, 81.8 mg/L; CD4+ T lymphocyte count, 80 cell/μL; CD8+ T lymphocyte count, 125 cell/μL; CA125, 50.08 U/mL; procalcitonin, 0.61 ng/mL. Tests for human immunodeficiency virus (HIV), (1,3)-β-D-glucan and galactomannan were negative. His autoimmune antibody levels were normal. Interferon gamma (IFN-γ) release assay (IGRA) was negative, and the PPD skin test was positive. The computed tomography findings of the multiple organs are shown in Figure 1. Multiple ring-enhancing low-density nodules in the head, neck, liver, and left psoas major muscle, and diffuse miliary nodules in both lungs were found. Peritoneal drainage fluid culture revealed *Escherichia coli* and *Enterococcus faecium.* The patient was diagnosed with complicated intra-abdominal infections and suspected disseminated TB. Imipenem, cilastatin, and vancomycin were administered, and a diagnostic first-line anti-tuberculosis treatment was administered. His condition continued to progress and he developed a perforated bowel. Puncture drainage of the psoas major and neck abscesses was performed, and the drainage fluid showed acid-fast staining and polymerase chain reaction assays for *M. tuberculosis* were both positive. The surgical slices were transferred from a local hospital and re-examined in our hospital, which indicated positive polymerase chain reaction results for *M. tuberculosis* DNA. The diagnosis of disseminated TB was confirmed. The anti-tuberculosis treatment was continued. The cultures grew nonresistant *M. tuberculosis* after 6 weeks. The patient's condition slowly improved. The timeline of the patient, with relevant data on episodes and interventions, is presented in Figure 2.

## 3 Discussion

Disseminated TB is a life-threatening condition with a 25% to 30% mortality rate.^[4]^

The diagnosis of disseminated TB is notoriously difficult, even for physicians familiar with TB. The patient was diagnosed nearly ten months after developing an abscess. There is still no reliable, simple, point-of-care test to definitively diagnose TB, although diagnostic methods have greatly improved in recent years.^[5]^ Clinicians still need to evaluate the disease in terms of its risk factors, clinical manifestations, systematic involvement, laboratory tests, and imaging findings.

The patient had no underlying diseases, but his CD4+ T cell count in the peripheral blood was less than 100 cells/μL. This may be an important reason for TB dissemination. Common risk factors for disseminated TB include HIV infection/acquired immunodeficiency syndrome and immunosuppression from other causes such as organ transplantation, alcoholism, diabetes mellitus, and malignancies.^[^4^,^^6^^]^ Our patient presented with a low CD4+T cell count without HIV infection, diabetes, or other immunodeficiency; thus, idiopathic CD4 lymphocytopenia (ICL) could not be ruled out.^[7]^ ICLs are often associated with infection, but it is not known whether infection triggers or its consequences lead to ICLs. Depletion of CD4+T cells is believed to drive the progression of TB among patients with HIV coinfection. The production of cytokines, such as IFN-γ and TNF-α, is essential for the control of mycobacterial infection.^[8]^ Lower CD4+ lymphocyte and unstimulated cytokine production were found in HIV-seronegative adults with previous extrapulmonary tuberculosis; a subtle abnormality in innate immune function might be related to the decreased ability to contain *M. tuberculosis.* ^[9]^ Another recent study suggested that latent TB infection could still be controlled in a subset of macaques even if pulmonary CD4+T cells were completely ablated, and CD8+T cells and B cells might correlate with TB control.^[10]^Further research on the immune roles of CD4+T cells, CD8+T cells, and B cells in the control of human TB is needed.

In terms of laboratory examinations, 2 main immune-based approaches, IGRA and tuberculin skin test (TST), were used to diagnose latent TB infection.^[11]^ The patient tested negative for IGRA and positive for TST. IGRA is more specific than TST because it does not cross-react with the Bacille Calmette-Guérin vaccine or mycobacteria in the environment.^[4]^ However, little is known about the usefulness of IGRA and TST in diagnosing disseminated TB. A previous study showed that patients with a positive tuberculin test were less likely to have disseminated TB, but a positive IGRA could not predict the odds of disseminated TB.^[12]^ Another previous study found that IGRA tests had a higher sensitivity in the diagnosis of TB than the TST with a 5 mm cutoff. Extrapulmonary TB is associated with a risk of false-negative IGRA results.^[13]^ The combination testing of IGRA and TST might be able to increase the diagnostic sensitivity and specificity. In addition, molecular diagnostic methods, such as MTBDRplus, GeneXpert, and whole genome sequencing (WGS), have been increasingly used in the diagnosis and characterization of TB.^[14]^ However, the cost of testing and challenges of laboratory technology and methods are major obstacles to the clinical application of WGS.^[^15^–^16^]^

Disseminated TB usually affects multiple sites. In recent years, with the development of imaging technologies, they have become important auxiliary adjuncts in the diagnostic evaluation of disseminated TB.^[17]^ They can not only help determine the involved organs or sites and obtain specimens for diagnosis, but also help clinicians make a suspicious diagnosis through imaging manifestations. The imaging findings of this patient suggested multiple abscesses with rim enhancement. A study also found that abscess or tuberculoma with rim enhancement at multiple sites was the radiological characterization of disseminated TB in patients with AIDS. Miliary patterns in the lung, liver, or spleen and widespread lymphadenopathy with rim enhancement are also important CT findings of disseminated TB.^[18]^ PET/CT has also recently been successfully used as an assessment tool for disseminated TB.^[19]^ However, another study suggested that multiple systemic disseminated TBs could mimic lymphoma on PET/CT.^[20]^ Although the imaging findings are nonspecific, disseminated TB should be considered first if 2 or more non-contiguous sites are indicated by imaging examinations.^[18]^

## 4 Conclusion

In conclusion, diagnosis of disseminated TB is often difficult. Before reliable evidence for the diagnosis is obtained, an imaging examination should be selected. Disseminated TB should be considered in patients with multisystem illnesses, especially in areas with a high incidence of TB, and diagnostic evidence should be continuously searched.

## Acknowledgment

The authors thank the nurses at the Center of Infectious Disease, West China Hospital, Sichuan University for their clinical assistance.

## Author contributions

**Conceptualization:** Junyan Qu, Fang He.

**Data curation:** Cejun Zhong, Huan Li.

**Funding acquisition:** Junyan Qu, Fang He.

**Project administration:** Huan Li, Fang He, Cejun Zhong.

**Supervision:** Junyan Qu, Fang He.

**Writing – original draft:** Huan Li, Junyan Qu.

**Writing – review & editing:** Fang He, Junyan Qu.
